# Supplementary material for: Integrative analysis of microRNA and mRNA expression profiles in fetal rat model with anorectal malformation
Source: PeerJ. 2018 Oct 24;6:e5774. doi: 10.7717/peerj.5774 (PMC6203938; doi:10.7717/peerj.5774)
Supplement: Supplemental Information 1 [file peerj-06-5774-s008.pdf]

## Supplymentary material 1

A B C

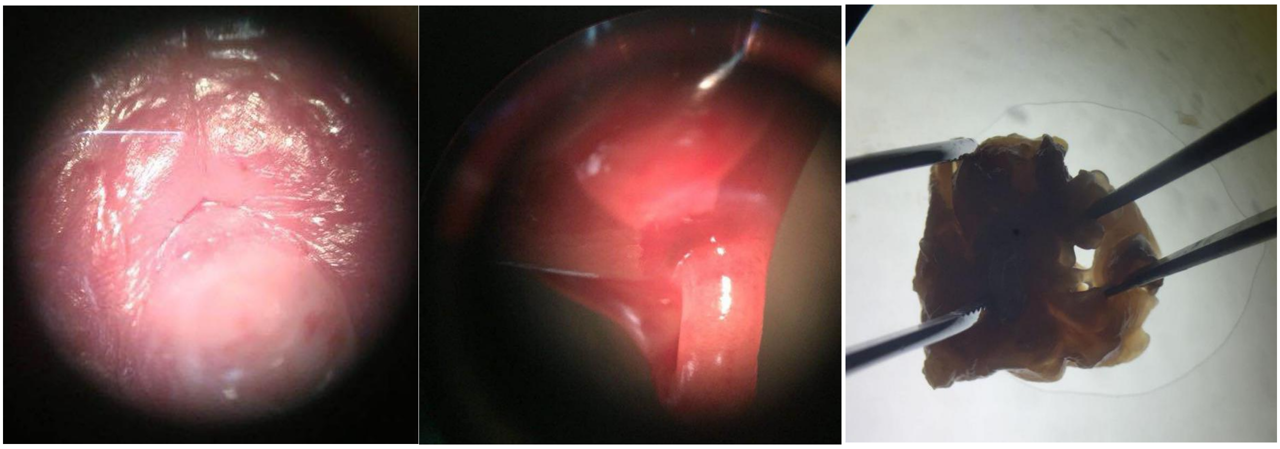

**A** The appearance of the anus of control E20 fetal rat under the stereomicroscope. **B** The appearance of the anus of ARM E20 fetal rat under the Stereomicroscope. **C** The hindgut tissue of fetal rats of E20
