# Supplementary material for: Integrative analysis of microRNA and mRNA expression profiles in fetal rat model with anorectal malformation
Source: PeerJ. 2018 Oct 24;6:e5774. doi: 10.7717/peerj.5774 (PMC6203938; doi:10.7717/peerj.5774)
Supplement: Supplemental Information 2 [file peerj-06-5774-s009.pdf]

Supplymentary material 2

Sample quality inspection report									
The detection results of sample									
NO.	Sample name	Concentrations (μg/μl)	A260/280	A260/230	Volume (μl)	Total weight (μg)	2100		Result
							28S/18S	RIN	
1	E20 control group-1	0.5665	2.17	2.07	20	11.33	2.3	6.1	C
2	E20 control group -2	0.4394	2.14	1.89	20	8.79	1.8	8.2	A
3	E20 control group -3	0.4914	2.11	1.88	20	9.83	1.6	7.6	A
4	E20 control group -4	0.2563	2.17	1.90	20	5.13	2.3	7.2	A
5	E20 control group -5	0.2663	2.17	2.12	20	5.33	1.7	8.9	A
6	E20 ARM group -1	0.2880	2.18	1.82	20	5.76	2.2	9.4	A
7	E20 ARM group -2	0.3759	2.14	1.88	20	7.52	2.3	9.1	A
8	E20 ARM group -3	0.8122	2.16	1.88	20	16.24	2.2	9.0	A
9	E20 ARM group -4	0.3879	2.15	1.91	20	7.76	2.2	9.5	A
10	E20 ARM group -5	0.6145	2.18	2.07	20	12.29	2.3	9.4	A

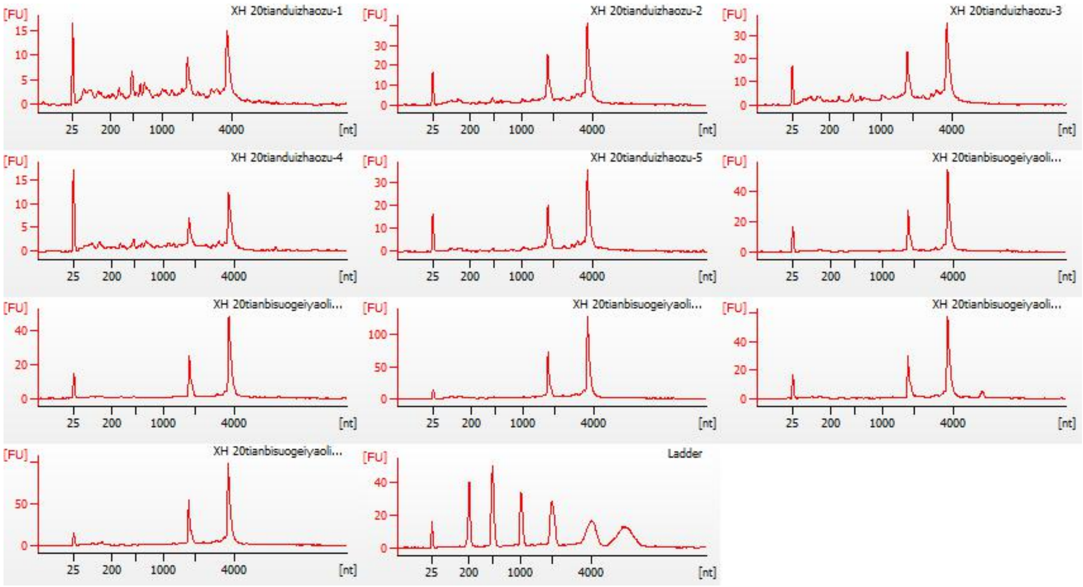

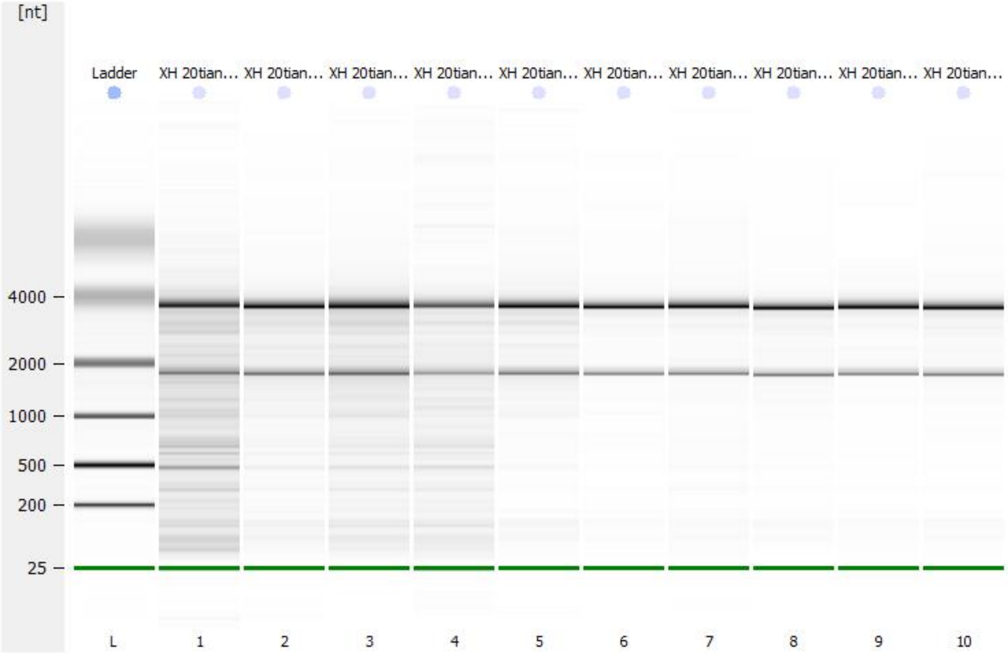

RNA quantification standard：

(1) Total RNA>=5μg;

(2) OD 260/280∈[1.8-2.2]

(3) Agilent Bioanalyzer 2100 inclusion criteria: A: **RIN>=7** and 28S/18S>=0.7, follow-up experiments can be carried out.

The control group sample 2, 3, 5 and ARM group sample 1, 4, 5 were included in the microarray analysis.
