# Supplementary material for: Integrative analysis of microRNA and mRNA expression profiles in fetal rat model with anorectal malformation
Source: PeerJ. 2018 Oct 24;6:e5774. doi: 10.7717/peerj.5774 (PMC6203938; doi:10.7717/peerj.5774)
Supplement: Supplemental Information 3 — U6 and GAPDH were used as the internal controls for miRNAs and mRNA, respectively. [file peerj-06-5774-s010.pdf]

Supplementary material. 3 Primers sequences for miRNAs and mRNAs

**Gene names**	**Forward** (5' - 3')	**Reverse** (3' - 5')
rno-miR-1-3p	GTGGAATGTAAAGAAGTGTGTA	TTCGCACTGGATACGACGGCCATGC
rno-miR-206-3p	TGGAATGTAAGGAAGTGTGTG	TTCGCACTGGATACGACGGCCATGC
rno-miR-3084a-3p	TTCTGCCAGTCCCCTTCAGA	TTCGCACTGGATACGACGGCCATGC
rno-miR-598-3p	TACGTCATCGTCGTCATCGTT	TTCGCACTGGATACGACGGCCATGC
Rno-miR-99b-3p	TAACACTGTCTGGTAACGATGT	TTCGCACTGGATACGACGGCCATGC
U6	CAAGGATGACACGCAAATTCG	TTCGCACTGGATACGACGGCCATGC
Wnt16	CTTCAAATCCCAAGCTGCAC	CTGTTCCCTATATGCCAACG
Cdh15	TCTCGGAGCAAGAGTCTG	TATGTCGTAGGCATCCTGG
Trim5	TATAACCTCATTCGGCCCATC	CAGGGTGCATTTCTTAAGC
Ccl7	CTGAAGCCCATCAGAAGTG	GTTGGTTTCTGTTCAGGCA
Fgf16	AACGACACCAGAAATTCACTCA	GGACATGGAGGGCAACTTA
GAPDH	CTCAGACACCATGGGGAAGGTGA	ATGATCTTGAGGCTGTTGTCATA

U6 and GAPDH were used as the internal controls for miRNAs and mRNA, respectively.
